# Plant identification credibility in ethnobotany: a closer look at Polish ethnographic studies

**DOI:** 10.1186/1746-4269-6-36

**Published:** 2010-12-17

**Authors:** Łukasz J Łuczaj

**Affiliations:** 1 The Wild Garden; Pietrusza Wola 86; 38-471 Wojaszówka; Poland

## Abstract

**Background:**

This paper is an attempt to estimate the percentage of erroneously identified taxa in ethnographic studies concerning the use of plants and to propose a code for recording credibility of identification in historical ethnobotany publications.

**Methods:**

A sample of Polish-language ethnobotanical literature (45 published sources from 1874-2005) and four collections of voucher specimens (from 1894-1975) were analyzed. Errors were detected in the publications by comparing the data with existing knowledge on the distribution of plant names and species ranges. The voucher specimens were re-examined.

A one-letter code was invented for quick identification of the credibility of data published in lists of species compiled from historical or ethnographic sources, according to the source of identification: voucher specimen, Latin binominal, botanical expert, obvious widespread name, folk name, mode of use, range, physical description or photograph. To test the use of the code an up-to-date list of wild food plants used in Poland was made.

**Results:**

A significant difference between the ratio of mistakes in the voucher specimen collections and the ratio of detectable mistakes in the studies without herbarium documentation was found. At least 2.3% of taxa in the publications were identified erroneously (mean rate was 6.2% per publication), and in half of these mistakes even the genus was not correct. As many as 10.0% of voucher specimens (on average 9.2% per collection) were originally erroneously identified, but three quarters of the identification mistakes remained within-genus.

The species of the genera *Thymus, Rumex *and *Rubus *were most often confused within the genus.

Not all of the invented credibility codes were used in the list of wild food plants, but they may be useful for other researchers. The most often used codes were the ones signifying identification by: voucher specimen, botanical expert and by a common name used throughout the country.

**Conclusions:**

The results of this study support the rigorous use of voucher specimens in ethnobotany, although they also reveal a relatively high percentage of misidentified taxa in the specimens studied.

The invented credibility coding system may become a useful tool for communication between historical ethnobotanists, particularly in creating larger databases.

## Background

One of the main problems ethnobotanists face when publishing their results is the possibility of a mistake in the identification of the studied taxa. Therefore securing voucher specimens is now standard procedure in ethnobotany [1-3], required by major journals and discussed in ethnobotany method manuals [e.g. [4,5]]. On the other hand the results of studies not documented by voucher specimens are still sometimes published, particularly in the field of historical ethnobotany, where not only is there a lack of voucher specimens, but often we have to hypothesize about the taxonomic position of certain species known only by their extinct folk/local names [6]. Ethnobotanists may include sources in their databases, which contain Latin binominals that come from reliable authors (preferably from professional botanists), but which are not confirmed by voucher specimens. This situation comes about because historical data are often too important to be discarded just on the basis of insufficient documentation [7].

It seems that no one has ever endeavored to estimate the possible percentage of mistakes in ethnobotanical publications. One of the very few authors who has dealt with the credibility of data in historical ethnobotany is Svanberg [8,9]. He presented a few examples of some so called "ghost data" - old and erroneous information, which has been repeated by subsequent authors. The importance of identification credibility in historical ethnobotany can be clearly shown by the study of Kufer *et al*. [10], who compared present use of plants by the Ch'orti' Maya from Guatemala with data gathered in the same population in the 1930s by Charles Wisdom. It turned out that some mistakes occurred in the former study, where a taxon was misidentified as belonging to a different family.

The quality of ethnobotanical information is increasingly discussed in a variety of contexts [11-13], for instance ethnobotanical databases [14,15]. For example in a database of ethnobotanical data on the Campania region in Italy [14], levels of certainty of identification were introduced (sure, unsure, etc.). Generally, the likelihood of a mistake in identification probably increases with the age of the studied publication/information. This happens for a variety of reasons, e.g. changing folk names or uses in time.

In order to analyze the issue of mistakes in plant identification we should look at the whole process of plant identification. With ethnobotanical data a few scenarios are most likely:

1. The plant was shown to the ethnobotanist by an informant.

1.1 The informant showed the wrong plant.

1.2 The informant showed the right plant.

1.2.1 The plant was not taken from the field and the identification was performed "from memory".

1.2.2 The plant was picked and used in the identification process.

   1.2.2.1 The plant was not preserved.

      1.2.2.1.1 A voucher specimen was not made.

      1.2.2.1.2 A voucher specimen was made later. from a plant, which according to the ethnobotanist's knowledge belongs to the same taxon.

   1.2.2.2 The plant was preserved as a voucher specimen.

2 The plant was not shown to the ethnobotanist.

2.1 The plant was named by the informant using a local name.

2.1.1 A scientific name was not assigned.

2.1.2 The scientific name was found/hypothesized using other ethnobotanical literature containing the same or similar folk names as used in the studied population.

2.1.3 The local name is identical or similar to an official 'scientific' name of a species and the plant was (often erroneously) identified by assuming that the local name referred to the same taxon.

2.2 The plant was named by the informant using its scientific name (and a local name).

2.3 The plant was identified by the ethnobotanist from a verbal description.

Obviously the ideal situation is 1.2.2.2, particularly if voucher specimens were shown/brought by more than one informant. However, different scenarios happen for a variety of reasons, of which the major three are:

1 the ignorance of the researcher,

2 the fact that the information may be published/recorded even if securing of a voucher specimen is not possible, because of the importance of studying the use of a taxon for the researcher,

3 the use of a plant is extinct and we have only historical records without voucher specimens.

In this study I would like to consider the problem of the credibility of ethnobotanical data in one country - Poland. Poland, like a few other European countries, has a rich 19 and 20^th ^century ethnographic literature concerning the traditional use of plants - for a bibliography see Klepacki's review [16]. As the Polish flora is relatively poor in plant species (it has approximately three thousand species), the concept of voucher specimens was difficult to understand, not only for ethnographers studying the traditional use of plants, but also for botanists, who were relatively sure of their identifications.

The first person who tried to verify the credibility of older ethnobotanical studies in Poland was Köhler in 1996 [17], who checked the identification of plants in Udziela's herbarium from the turn of the 19^th ^and 20^th ^century. A few years earlier Radwańska-Paryska [18] re-examined the herbarium of an 18^th ^century monk, Brother Cyprian, containing Slovak and Polish plant names from the Pieniny and Tatra mountain ranges bordering the two countries. Later, the author of this paper (ŁŁ) published an article on the taxonomic issues concerning the quality of the data and mistakes in the identification of taxa in ethnobotanical studies in Poland [19].

The aim of this article is to extend the investigations of the previous work [19], in particular:

1. To quantify a possible percentage of taxonomic errors in publications from this field.

2. To propose a standard of coding the credibility of identification of scientific names in ethnobotanical publications, and test its usefulness by making a list of edible plants used in Poland.

## Methods

A sample of Polish-language ethnobotanical literature consisting of 45 published sources [20-64] (Table 1) and four voucher specimen collections were analyzed (Table 2). The analyzed publications consisted of a large proportion of Polish-language ethnographic publications with ethnobotanical content, which contained lists of regionally used plants including at least one Latin name. All such papers available to the author were taken into account. Most of the analyzed sources deal with either wild food plants (reviewed in the Journal of Ethnobiology and Ethnomedicine in 2007 [65]) or medicinal plants. Papers without Latin binominals or monographs on the use of single species were not included. Maurizio's [66] and Moszyński's [67,68] major works were not taken into account, as they are syntheses concerning the whole of northern Eurasia (the former author) or all Slavs (the latter). Lists of plant names and databases compiled mainly on the basis of other published sources were not included either [e.g. [69-71]]. The analyzed publications usually concern studies from the present area of Poland and in a few cases - western Belarus [44,45,47], western Ukraine [37] and Lithuania [56]. The publications from these countries were included in the analyses as they were written by Polish ethnographers working close to the present area of Poland, within its former, broader territory from before World War II.

**Table 1:** Literature sources [20-64] where the level of botanical mistakes was assessed using comparative methods (using the present knowledge of species ranges and the distribution of folk names).

Author's name	Reference Number	Year	Main topic	No. of taxa with Latin names	Errors
Bohdanowicz	[20]	1996	food	10	2
Chętnik	[21]	1936	food	14	0
Dekowski	[22]	1968	food	35	0
Dekowski	[23]	1973	foraging	38	0
Dydowiczowa	[24]	1964	foraging	44	0
Eljasz-Radzikowski	[25]	1897	general ethnographic	18	0
Gajkowa	[26]	1947	general ethnographic	4	3
Gawełek	[27]	1910	ethnomedicine	36	0
Gustawicz	[28]	1904	general ethnographic	18	0
Henslowa	[29]	1962	selected edible taxa	12	0
Janicka-Krzywda	[30]	2004	food	5	1
Jostowa	[31]	1954	food	1	0
Kantor	[32]	1907	general ethnogr.	46	5
Kolberg	[33]	1962 (1874)	general ethnogr.	54	1
Kolberg	[34]	1962 (1882)	general ethnogr.	35	1
Kolberg	[35]	1962 (1891)	general ethnogr.	75	0
Kolberg	[36]	1973	general ethnogr.	6	0
Kolberg	[37]	1963 (1888)	general ethnogr.	22	0
Kolberg	[38]	1968	general ethnogr.	29	0
Libera, Paluch	[39]	1993	ethnomedicine	98	0
Łęga	[40]	1961	general ethnogr.	2	0
Malicki	[41]	1971	foraging	20	0
Ochrymowicz	[42]	1900	beliefs about herbs	52	1
Oczykowski	[43]	1896	ethnomedicine	10	1
Orzeszkowa	[44]	1888	ethnomedicine and beliefs	69	0
Orzeszkowa	[45]	1891	ethnomedicine and beliefs	51	0
Paluch	[46]	1984	ethnomedicine	176	0
Pietkiewicz	[47]	1928	material culture	23	0
Plichta	[48]	1891	ethnomedicine	8	0
Ruszel	[49]	2004	ethnographic dictionary	85	8
Siarkowski	[50]	1890	ethnomedicine	17	0
Siarkowski	[51]	1891	ethnomedicine	5	1
Sulisz	[52]	1906	general ethnogr.	11	3
Sulisz	[53]	1906	general	8	4
Szot-Radziszewska	[54]	2005	ethnomedicine	129	4
Szromba-Rysowa	[55]	1966	foraging	63	2
Szukiewicz	[56]	1903	folk beliefs	5	0
Szulczewski	[57]	1996	general ethnogr.	>100	0
Szychowska-Boebel	[58]	1972	ethnomedicine	95	1
Szychowska-Boebel	[59]	1978	ethnomedicine	49	1
Tylkowa	[60]	1988	ethnomedicine	81	4
Wawrzeniecki	[61]	1916	ritual plants	14	2
Weryho	[62]	1888	ethnomedicine	31	1
Wysłouchowa	[63]	1896	general ethnogr.	38	0
Udziela	[64]	1931	ethnomedicine and beliefs	141	0

**Table 2:** Voucher specimen collections analyzed.

Author's name	Publication place of original names	Publication place of corrected names	No. of voucher specimens analyzed	No. of errors
Udziela	[61]	[17]	119	8
Orzeszkowa	[44,45]	partly in [77]	129	8
Gajek	unpublished	[79]	196	28
Szychowska-Boebel	[59]	-	21	2

The total number of identified plant taxa was recorded for each publication, as well as the number of taxa which were presumably identified erroneously. A reference to a species from one publication and each herbarium specimen were later referred to as a use-report, a term, which, although mainly applied to indicate a plant-use mentioned by a given informant [72], in this case can be used with a publication as a unit. This way of treating a literature citation as one use-report is used in ethnobotanical studies, which review earlier publications, where the number of informants and informant consensus is not given. For example this approach was used by Leonti et al. [73] to analyze the influence of the 16^th ^century herbal of Matthioli on present day ethnobotanical knowledge in Campania (Italy), and in reviews of edible plants of Spain [74].

The following methods of identifying errors were used:

- For wild taxa the distribution was checked in the atlas of the distribution of Polish vascular plants [75] - if the species did not occur in the geobotanical region (*kraina geobotaniczna *as mapped by Matuszkiewicz [76]) of the publication, an error was assumed.

- Some taxa were widely used under one name and their 'identity' is obvious but a different Latin name had been erroneously assigned to this folk taxon. For example in one publication *szałwia - Salvia officinalis *was named *S. pratensis*, although the description of the plant without doubt refers to the former.

The second part of the study dealt with the re-examination of voucher specimens (Table 2). The voucher specimen collections for ethnobotanical data are extremely rare in Poland and so far only four such herbariums have been found:

1. The documentation of Udziela's study [61] of medicinal and ritual plants of the Kraków area, stored in the Herbarium of the Institute of Botany of the Polish Academy of Sciences in Kraków (KRAM). The whole collection (119 specimens) was already previously checked by Köhler [17] but in 2010 I reexamined the collection. The specimens probably come from 1894-99 when Udziela collected his field data [17].

2. The documentation of Orzeszkowa's ethnobotanical study from the river Niemen region (now western Belarus) published in a few parts in the periodical *Wisła *between 1888 and 1891 [e.g. [44,45]] stored in the archive of the Poznańskie Towarzystwo Przyjaciół Nauk society in Poznań. The detailed description of this herbarium was published by Kielak [77]. Kielak's book contains colour photographs of around half of the voucher specimens in the archive (129 specimens out of 280). Plants were re-identified using photographs from this book.

3. The archives of the Polish Ethnographic Atlas study of wild edible plants from 1948-49 and medicinal plants from 1949-50 [78]. The herbarium (as a part of the field questionnaires) is stored in the office of the Polish Ethnographic Atlas in Cieszyn (University of Silesia) but formally belongs to the Polish Folklore Society in Wrocław. For this study 196 questionnaires (concerning edible plants) containing herbarium specimens, identified with Latin names, were used. The person who identified them is not recorded, the name of the Department of Plant Systematics and Geography of the University of Curie-Skłodowska in Lublin is printed as the identifying institution. The content of these questionnaires was published in 2008 with identifications already corrected by Łuczaj [79] - however in this study the original identifications were analyzed with reference to the kinds of errors that were made. The archive contains a few hundred more voucher specimens but they were not included in this study as they were only recently rediscovered and have not been analyzed in detail.

4. The herbarium of Szychowska-Boebel, stored in the archive of the Ethnographic Museum in Toruń. It is a documentation of her studies of ethnomedicinal plants in the village of Wiele in Eastern Pomerania in 1975 [59]. It contains 43 specimens, including 21 identified taxa.

Both in publications and voucher specimen collections, only taxonomic errors were taken into account. Spelling mistakes were not included, nor were cases where the author was cautious and identified only the genus (for example *Equisetum *sp. instead of *Equisetum hyemale*). However the cases when only one species was reported in the literature as used in the area, though we have firm evidence that a larger number of closely related species was/is utilized were also treated as errors (inaccuracies), for example, a passage like: "blackberries (*Rubus caesius*) are used as food", as "*Rubus caesius" *should be replaced by "*Rubus *subgenus *Rubus" *or "*Rubus *spp."

The author set up a code of credibility for presentation of historical ethnobotanical data in tables:

H - confirmed by (a) voucher specimen(s),

A - confirmed by authority (expert botanist),

O - obvious common name universally used in a large area,

L - highly probable Latin name or a binominal scientific name used in the language of a given country corresponding to a Latin name, given by non-botanist,

N - identified using comparative analysis of folk names,

M - identified using data on the species' mode of use (in case of unusual species/uses),

D - identified using physical description of species,

R - identified with the help of the data of a species range or/and habitat,

U - highly uncertain (should be combined with another code),

P - identified using pictures (photographs or drawings).

The usefulness of such a code was tested by compiling an up-to-date list of wild food plants used in Poland from the 19^th ^to 21^st ^century (within the present territory, excluding the German population pre-1939). The list was based on the review of edible plants of Poland [65] and amended by recent publications by Łuczaj [79-81] and Pirożnikow [82,83] bringing more data on the subject.

## Results

Forty-six identification mistakes were detected both in the published material using comparative methods (Tables 1 and 3) and in the voucher specimens (Tables 2, 4 and 5). This constitutes 2.3% of the analyzed use-reports for the former set of data and 10.0% of voucher specimens. The mean mistakes rates per publication differ significantly between the two sets of data (Mann-Whitney U test, U = 35.5, P (exact version) = 0.032, P (Monte Carlo version) = 0.022), they were 6.2% and 9.2% respectively.

**Table 3:** Errors detected in the studied publications, assessed using comparative methods.

Author	Name in the publication (with original spelling)	The correct name	Type of mistake/inaccuracy
Bohdanowicz [20]	*Origanum vulgare*	*Chenopodium*sp.	L
Bohdanowicz [20]	*Carduus*	*Cirsium *sp.	L
Gajkowa [26]	*cuminum cyminum*	*Carum carvi *L.	L
Gajkowa [26]	*panicum miliaceum*	*Echinochloa crus-galli *(L.)P.Beauv.	L
Gajkowa [26]	*atriplex hortense*	*Chenopodium & Atriplex *spp.	L
Janicka-Krzywda [30]	*Carlina vulgaris*	*Carlina acaulis *L.	?
Kantor [32]	*Geranium*	*Dahlia *sp.	?
Kantor [32]	*Iris*	*Lilium *sp.?	?
Kantor [32]	*Salsola*	?	?
Kantor [32]	*Selinum carvifolia*	*Carum carvi *L.	I
Kantor [32]	*Sesleria coerulea*	*Sesleria sadlerana *Janka ssp. *tatrae *(Degen) Deyl?	I
Kolberg [33]	*Hippophae rhamnoides*	*Salix *sp.	L
Kolberg [34]	*Helleborus albus*	*Veratrum *sp.*? Vincetoxicum hirundinaria *Medik.?	L
Ochrymowicz [42]	*Iris germanica*	*Iris *sp. or *Eupatorium cannabinum *L.	?
Oczykowski [43]	*Rumex hydrolapathum*	some other *Rumex *spp.	I
Ruszel [49]	*Plantago major*	*Plantago *spp.	S (the name refers to all the *Plantago *species)
Ruszel [49]	*Helleborus viridis*	*Veratrum lobelianum *Bernh.*?*	L
Ruszel [49]	*Carum carvi*	*Glechoma hederacea *L. s.l.	?
Ruszel [49]	*Thymus serpyllum*	*Thymus serpyllum *L. em. Fr. &*Th. pulegioides *L.	S (both species are used)
Ruszel [49]	*Tilia cordata*	*Tilia cordata *Mill. &*T. platyphyllos *Scop.	S (both species are used equally frequently)
Ruszel [49]	*Rubus plicatus*	*Rubus *subgenus *Rubus *spp.	S (there are a few dozen species of *Rubus *in this area, *R. plicatus *is not the most frequent [84]
Ruszel [49]	*Carduus*	*Cirsium & Carduus *spp.	L
Ruszel [49]	*Rumex hydrolapathum*	*Rumex *spp. mainly *R. obtusifolius *L.	I
Siarkowski [51]	*Thymus serpyllum*	*Thymus *spp.	S
Sulisz [52]	*Origanum vulgare*	*Chenopodium *sp.	L
Sulisz [52]	*Thymus vulgaris*	*Thymus serpyllum *L. em. Fr. or *Th. pulegioides *L.	I
Sulisz [52]	*Matricaria Chamomilla*	*Tanacetum parthenium *(L.) Sch.Bip.	I
Sulisz [53]	*Acorus calamus*	*Calamagrostis epigejos *(L.)Roth	?
Sulisz [53]	*Rhamnus cathartica*	*Staphylea pinnata *L.	?
Sulisz [53]	*Galium cruciata*	*Euonymus europaeus *L.*/Rhamnus cathartica *L.*?*	? (L?)
Sulisz [53]	*Ledum palustre*	the term *bagnięta *was used erroneously as it refers to any wooden branches	L
Szot-Radziszewska [54]	*Cicuta virosa*	*Solanaceae*, probably *Hyoscyamus niger *L.	L
Szot-Radziszewska [54]	*Thymus serpyllum*	*Thymus *spp.	S
Szot-Radziszewska [54]	*Salvia pratensis*	*Salvia officinalis *L.	L
Szot-Radziszewska [54]	*Papaver rhoeas*	*Papaver officinalis *L.	L
Szromba-Rysowa [55]	*Rubus caesius*	*Rubus *subgenus *Rubus *spp.	S (other *Rubus *spp. are used more frequently)
Szromba-Rysowa [55]	*Carduus *sp.	*Cirsium & Carduus *spp.	L
Szychowska-Boebel [59]	*Crataegus oxyacantha *L.	*Crataegus *spp.	S (*Crataegus monogyna *is more frequent)
Szychowska-Boebel [59]	*Crataegus oxyacantha *L.	*Crataegus *spp.	S (as above)
Tylkowa [60]	*Sonchus olearceus *L.	*Taraxacum *sp.	L
Tylkowa [60]	*Rubus plicatus *L.	*Rubus *spp.	S (there are a few dozen species of *Rubus *in this area, *R. plicatus *is not the most frequent [84]
Tylkowa [60]	*Malva alcea *L.	*Alcea rosea *L.	L
Tylkowa [60]	*Thymus serpyllum *L.	*Thymus pulegioides *L.	S - *T. serpyllum *does not occur in the region, the other species is commonly used
Wawrzeniecki [61]	*Thymus vulgaris*	*Thymus *spp.	I
Wawrzeniecki [61]	*Urtica urens*	*Urtica dioica *L. &* U. urens *L.	S
Weryho [62]	*Vinca major*	*Vinca minor *L.	?

L - wrong Latin name given by a researcher who looked the plant up in a guide using the local name as if it was an official name; I - other kind of wrong identification of a species; S - oversimplification/inaccuracy - one name given because more than one species from the genus is known under the same folk name, and the names are used with at least equal frequency, and in the same way, in the local area.

**Table 4:** Errors detected in the voucher specimen collections.

Collector	Name in the publication	The correct name
Orzeszkowa	*Anchusa arvensis*	*?*
Orzeszkowa	*Thymus serpyllum*	*Thymus pulegioides *L.
Orzeszkowa	*Anchusa arvensis*	*Anchusa officinalis *L.
Orzeszkowa	*Asarum europaeum*	*Hepatica nobilis *L.
Orzeszkowa	*Sium latifolium*	*Cicuta virosa *L.
Orzeszkowa	*Ranunculus sceleratus*	*Ranunculus flammula *L.
Orzeszkowa	*Ranunculus flammula*	*Ranunculus sceleratus *L.
Orzeszkowa	*Lamium maculatum*	*Lamiaceae *but not *Lamium*
Udziela	*Arabis arenosa*	*Epilobium adenocaulon *Hasskn.
Udziela	*Daucus carota*	*Pimpinella saxifraga *L.
Udziela	*Inula germanica*	*Inula britannica *L.
Udziela	*Lappa maior (=Arctium lappa)*	*Arctium tomentosum *Mill.
Udziela	*Marrubium vulgare*	*Nepeta cataria *L.
Udziela	*Mentha piperita*	*Mentha *cfr *verticillata *L.
Udziela	*Thymus serpyllum*	*Thymus pulegioides *L.
Udziela	*Tilia grandiflora*	*Tilia cordata *Mill.
PAE	*Betula alba*	*Betula pubescens *Ehrh.
PAE	*Carlina vulgaris*	*Cirsium arvense *(L.) Scop.
PAE	*Echium vulgare*	*Symphytum officinale *L.
PAE	*Hypericum perforatum*	*Vaccinium uliginosum *L.
PAE	*Malva neglecta *(x3)	*Malva sylvestris *L.
PAE	*Mentha cfr. aquatica*	*Mentha longifolia *(L.)Huds.
PAE	*Mentha piperita*	*Mentha longifolia *(L.)Huds.
PAE	*Polygonum bistorta*	*Rumex acetosa *L.
PAE	*Polygonum convolvulus*	*Convolvulus arvensis *L.
PAE	*Polygonum mite *(x2)	*Polygonum lapathifolium *L. s.l. (including *P. tomentosum *Schrank)
PAE	*Ribes rubrum *(x2)	*Ribes spicatum *Robson
PAE	*Rosa canina *(x3)	*Rosa *sp.
PAE	*Rubus hirtus*	*Rubus *sp.
PAE	*Rubus hirtus*	*Rubus caesius *L.
PAE	*Rubus saxatilis*	*Rubus caesius *L.
PAE	*Rumex acetosella*	*R. thyrsiforus *Fing.
PAE	*Rumex acetosella L *(x 2)	*Rumex acetosa *L.
PAE	*Thymus serpyllum *(x2)	*Thymus pulegioides *L.
PAE	*Trifolium medium*	*Trifolium repens *L. and *T. pratense *L.
PAE	*Trifolium medium*	*Trifolium pratense *L.
Szychowska-Boebel	*Trifolium arvense *L.	*Trifolium pratense *L.
Szychowska-Boebel	*Trifolium arvense *L.	*Trifolium repens *L.

PAE - Polish Ethnographic Atlas (specimens collected by numerous researchers)

**Table 5:** Comparison of error rates in the studied sources

Type of study	Literature	Voucher specimens
Number of publications/herbariums	45	4

No. of use-reports/specimens	1983	459

No. of errors detected	46	46

Average rate of mistakes per publication/source	6.2	9.2

Percentage of errors detected	2.3	10.0

Types of errors:	Number of taxa (Percentage given in parentheses)	

wrong genus	22 (48%)	11 (24%)

wrong species within the same genus	7 (15%)	29 (63%)

more species from the same genus are actually used in the area	16 (35%)	-

the identification is too detailed (the voucher specimen is in bad condition - it should have been identified only to the genus level)	-	5 (11%)

The comparative method revealed a relatively large number of mistakes in a few publications, both older [26,32,52,53] and new ones [49,54,60], however no or single mistakes were found in most sources.

There was no correlation between the year of publication and the percentage of errors in the species list (Pearson correlation coefficient, *r *= -0.004, P = 0.98, Kolberg's postmortem publications were assigned to his death date of 1890). Longer lists of plants had slightly lower error rates (the correlation between the number of Latin binominals in a list and the percentage of errors in it was *r = *-0.28, P = 0.060).

The mistakes concerned a variety of taxa but only a few taxa were mistaken more than twice: *Thymus*, ten times (e.g *Thymus serpyllum *confused with *Thymus pulegioides *or *T. vulgaris*), *Rubus *(six), *Rumex *(six), *Cirsium*, *Trifolium *(both four), *Chenopodium/Atriplex, Malva *and *Mentha *(three each). When the taxa from two families were confused this usually happened because of two similar folk/scientific names (e.g. *Chenopodium - *'lebioda', *Origanum vulgare *- 'lebiodka'; *Hippophae rhamnoides *-' rokitnik', narrow leaved *Salix *spp. - 'rokita', etc.), which suggests that the author looked up Latin names in a scientific key without illustrations. This kind of error was the commonest type of mistake (eighteen out of thirty-six errors where a possible reason for the error was identified). The second commonest type (twelve cases) were simplifications and inaccuracies - such as reporting the use of only one species when more species from the same genus were used at least as frequently (Table 3).

In the list of edible plants of Poland (Table 6) 39% of 192 use-reports are confirmed by voucher specimens (code H), 30% by scholars with reliable botanical expertise (code A), 13% using folk names known widely throughout the country and 11% by scientific names with unknown reliability (L). Only ten out of 192 were identified using folk names (N) and four by comparing species ranges (R; with help of other data, e.g. folk names). None of the species were identified by only using a physical description from literature (D), pictures (P) or mode of use (M). In ten cases the code U (uncertain) was used.

**Table 6:** The list of wild food plants used in Poland since the 19th century.

Species	Family	Method of Identification	Source	Parts Used	Mode of Use
*Acer platanoides *L.	*Aceraceae*	H	1, 2, 5	sap	raw and fermented
		A	1	cambium	raw
		A	1	fruits	raw
		A	1	opening leaf buds	fermented
		A	1, 5	leaves	under baking bread
*Acer pseudoplatanus *L.		A	1, 2	sap	raw
		A	1	leaf buds	raw, ff
*Aegopodium podagraria *L.	*Apiaceae*	A	3, 5, 6	young leaves	soup
*Carum carvi *L.		H	1, 2, 5	seeds	spice
		A	1	young plants	soup
*Daucus carota *L.		A	5, 6	roots, leaves, fruits	soup, spice
*Heracleum sphondylium *L.		H	1, 2, 3, 5, 6	leaves and flowering stalks	soup
*Pastinaca sativa *L.		O	1, 2, 6	roots	cooked foods
*Acorus calamus *L.	*Araceae*	H	1, 2, 5	inner parts of stems	raw
		H	1, 5	leaves	under baking bread
*Achillea millefolium *L.	*Asteraceae*	A	4, 5, 6	leaves	raw and as spice
*Arctium *sp.		A	3	leaf stalks	lacto-fermented
		A	6	roots	boiled
*Artemisia absinthium *L.		O	6	leaves	spice for meat
*Bellis perennis *L.		N	2	unspecified	unspecified
		A	5, 6	flowers	raw
*Carlina acaulis *L.		H	1, 2, 3	receptacles, roots	unknown
*Carlina vulgaris *L.		U	1	unspecified parts	unkown
*Centaurea cyanus *L.		H	1	petals	fermented drink
*Chamomilla recutita *(L.)Rauschert		L	2	shoots	infusion
*Cichorium intybus *L.		U	1, 6	leaves	boiled (ff), raw
		L	1, 5, 6	roots	coffee surrogate
*Cirsium oleraceum *Scop.		A	1, 3, 4	leaves, roots	boiled, ff
*Cirsium rivulare *All.		H	1, 2, 3, 4	leaves	boiled, ff
*Cirsium arvense *(L.) Scop.		H	2, 3, 4	leaves, stalks	boiled, ff
*Sonchus arvensis *L.		LU	1, 3	green parts	raw
*Taraxacum *sp. pl.		A	1, 5	inflorescences	syrup, wine
		H	1, 2, 5	leaves	raw, boiled
*Tragopogon pratensis *L. s.l.		H	2	stalks	raw
*Tussilago farfara *L.		A	1	leaves	boiled, ff
*Berberis vulgaris *L.	*Berberidaceae*	H	1, 2, 3	fruits	raw, preserves
*Alnus *sp.	*Betulaceae*	O	2		
*Betula pendula *Roth *& Betula pubescens *Ehrh.		H	1, 2, 5	sap	raw or fermented
		H	1	leaf buds	fermented
		H	1, 2	cambium	flour, ff
*Anchusa arvensis *(L.) M.Bieb.	*Boraginaceae*	A	3	shoots	boiled, ff
*Echium vulgare *L.		A	6	flowers	nectar sucked
*Pulmonaria obscura *L.		AR	1	leaves	boiled, ff
*Symphytum officinale *L.		H	1, 2, 3	leaves	boiled, ff
		H	2, 6	flowers	nectar sucked
		A	6	roots	boiled (ff?)
*Armoracia rusticana *P.Gaertn., B.Mey,&Scherb.	*Brassicaceae*	H	1, 2, 5	roots	spice
		A	1	leaves	under baking bread or as spice
*Capsella bursa-pastoris *(L.)Medik.		A	1, 2	fruits	raw
		A	5, 6	whole plant?	boiled
*Cardamine amara *L.		RU	3	leaves	raw
*Cardamine pratensis *L.		A	3	leaves	ff
*Raphanus raphanistrum *L.		H	1, 2, 3, 4	leaves	boiled, ff
*Sinapis arvensis *L.		H	1, 2, 3, 4	leaves	boiled, ff
*Campanula persicifolia *L.	*Campanulaceae*	L	1, 6	flowers	raw
*Phyteuma spicatum *L.		L	1	roots	unspecified
*Humulus lupulus *L.	*Cannabaceae*	O	1, 2, 5	inflorescences and fruits	beer, mead, bread
		O	1	probably shoots	ff
*Sambucus nigra *L.	*Caprifoliaceae*	H	1, 2, 3, 5, 6	fruits	boiled: wine, jam, soup, rarely raw
		O	3, 5	flowers	fried in batter or preserves
*Viburnum opulus *L.		A	1, 2, 5	fruits	boiled: wine, juice, jam
*Silene vulgaris *(Moench) Garcke	*Caryophyllaceae*	A	3	shoots	boiled, ff
*Stellaria media *(L.) Vill.		N	3	shoots	boiled, ff
*Euonymus verrucosus *Scop.	*Celastraceae*	A	5	fruits	added to wine?
*Atriplex patula *L.	*Chenopodiaceae*	L?	1	leaves	boiled, fried
*Chenopodium album *L.		H	1, 2, 3, 5	leaves	boiled, fried
*Chenopodium bonus-henricus *L.		A	1, 3	leaves	boiled, fried
*Chenopodium hybridum *L.		A	3	leaves	boiled, fried
*Chenopodium polyspermum *L.		A	3	leaves	boiled, fried
*Chenopodium polyspermum *L.		A	3	leaves	boiled, fried
*Convolvulus arvensis *L.	*Convolvulaceae*	H	1, 2, 3	shoots	boiled, ff
*Carpinus betulus *L.	*Corylaceae*	H	1	sap	raw
*Corylus avellana *L.		H	1, 3	inflorescences, leaves	ff, mainly for flour
		H	1, 2, 3, 5	fruits	raw and in cakes
*Juniperus communis *L.	*Cupressaceae*	O	1, 2, 5	pseudofruits	spice, beer, snack
*Scirpus sylvaticus *L.	*Cyperaceae*	A	1, 6	inner parts of young shoots	raw
*Pteridium aquilinum *L.	*Dennstaedtiaceae*	LU	1	rhizomes	unspecified, ff
*Empetrum nigrum *L.	*Empetraceae*	A	1	fruits	unspecified
*Equisetum arvense *L.	*Equisetaceae*	H	1, 2, 6	strobils	raw, cooked
		A	2, 6	bulbils	raw
*Calluna vulgaris *(L.) Hull ON	*Ericaceae*	L	1	seeds	bread, ff
*Vaccinium myrtillus *L.		H	1, 2, 3	fruit	raw, boiled
*Vaccinium oxycoccos *L.		H	1, 2, 3, 5	fruit	raw or in preserves
*Vaccinium vitis-idaea *L.		H	1, 2, 3, 5	fruit	raw or in preserves
*Vaccinium uliginosum *L.		H	1, 2, 3, 5		
*Euphorbia peplus *L.	*Euphorbiaceae*	L	1	whole plant	boiled, ff
*Astragalus glycyphyllos *L.	*Fabaceae*	A	5, 6	stalks	raw
*Medicago lupulina *L.		A	6	thickened parts of the roots	raw
*Robinia pseudoacacia *L.		H	2	flowers	raw, jams
*Trifolium pratense *L*., T. repens *L.*, T. montanum *L.		H	1, 2	inflorescences	nectar sucked or dried for baking bread
*Vicia *sp. pl.		L	1, 2	seeds	flour for bread, ff
*Fagus sylvatica *L.	*Fagaceae*	H	1, 3	fruits	raw or baked, oil
*Quercus robur *L. *& Q. petraea *Mattuschka (Liebl.)		H	1	fruits	flour (ff), coffee surrogate
*Ribes alpinum *L.	*Grossulariaceae*	A	5	fruits	raw (rarely)
*Ribes alpinum *L. or *R. petraeum *Wulfen		NR	1	fruits	raw
*Ribes nigrum *L.		H	1, 2, 6	fruits	raw, jams
		H	1, 6	leaves	spice
*Ribes spicatum *Robson		H	2, 6	fruits	raw, jams
*Ribes uva-crispa *L.		O	2, 3	fruits	raw
*Stratiotes aloides *L.	*Hydrocharitaceae*	H	2	leaves, roots	boiled, ff
*Dracocephalum ruyschiana *L.	*Lamiaceae*	A	5	flowers	nectar sucked
*Galeopsis *sp.		A	1	leaves	boiled, ff
*Glechoma hederacea *L. s.l.		H	1, 2, 3	leaves	spice
*Lamium *sp pl. (mainly *L. album *L.)		A	3	shoots	boiled
*Lamium purpureum *L.		A	5	shoots	boiled
*Melittis melisophyllum *L.		LU	1	leaves	unspecified, ff; liquors
		A	6	flowers	nectar sucked
*Mentha arvensis *L.		H	1, 6	leaves	spice, infusions, raw
*Mentha longifolia *(L.)Hudson		H	1	leaves	spice
*Origanum vulgare *L.		A	1	flowering tops	beer condiment
*Prunella vulgaris *L.		N	4	shoots	boiled, ff
*Stachys palustris *L.		H	2, 3	rhizomes	raw, boiled
*Thymus pulegioides *L.		H	l	flowering tops	spice, teas
*Thymus serpyllum *L.		H	1, 5	flowering tops	spice, teas
*Lemna minor *L.		L	1	leaves	fried, ff
*Allium ursinum *L.	*Liliaceae*	LU	1	roots	spice
		O	1, 5, 6	leaves	raw
*Allium *sp.		N	2	?	?
*Maianthemum bifolium *(L.) F. W. Schmidt		H	1, 3	fruits	raw, wine
*Viscum album *L.	*Loranthaceae*	H	2	fruits	raw
*Malva neglecta *Wallr.	*Malvaceae*	H	1, 2	leaves	boiled
		H	1, 2, 5	immature fruits	raw
*Malva sylvestris *L.		H	1, 2	leaves	boiled
		H	1, 2	immature fruits	raw
*Fraxinus excelsior *L.	*Oleaceae*	O	5, 6	fruits	boiled, ff
*Oenothera *sp.	*Onagraceae*	A	6	roots	boiled
*Oxalis acetosella *L.	*Oxalidaceae*	H	1, 2, 3, 5, 6	leaves	raw, cooked
*Oxalis stricta *L. s.l.		H	1, 2	leaves	raw, cooked
*Papaver rhoeas *L.	*Papaveraceae*	N	2, 5	seeds	unspecified
*Abies alba *Mill.	*Pinaceae*	O	1	young shoots	syrup
*Picea abies *(L.) H. Karst.		O	1	young shoots	raw, syrup
		O	1	male inflorescences	raw
		O	1	young cones	raw
		O	2	cambium	ff
*Pinus cembra *L.		O	1	male inflorescences	raw
		O	1	seeds	raw
*Pinus sylvestris *L.		O	5	young vegetative and generative shoots	raw, syrup
*Plantago lanceolata *L.	*Plantaginaceae*	L	1, 2, 6	leaves	boiled, raw
*Bromus secalinus *L.	*Poaceae*	A	1, 2, 3	seeds	ground for flour, ff
*Dactylis glomerata *L.		A	1	stem base	raw
*Elymus repens *(L.) Gould		H	1	rhizomes	ground for flour
*Festuca pratensis *L.		LU	1	seeds	unspecified
*Glyceria fluitans *(L.) R. Br.		A	1	seeds	seeds, boiled or for flour
*Glyceria plicata *Fries		L	1	seeds	seeds, boiled or for flour
*Phleum pratense *L.		N	2	seeds	for flour, ff
*Setaria pumila *(Poir.) Schult. or/and *Setaria viridis *(L.) P.Beauv.		NR	3	seeds	boiled
*Fallopia convolvulus *(L.) AA.Löve	*Polygonaceae*	H	3	shoots	boiled, ff
*Polygonum hydropiper *L.		A	5	leaves	raw
*Polygonum lapathifolium *L. ssp. *pallidum*		H	1	shoots	fried, ff
*Rumex acetosa *L.		H	1, 3, 5	leaves	raw, cooked
*Rumex acetosella *L.		H	1, 3	leaves	raw, cooked
*Rumex crispus *L.		A	6	leaves	cooked
*Rumex crispus *L. or *R. obtusifolius *L.		A	5, 6	fruit	boiled, ff
*Rumex hydrolapathum *Huds.		A	6	leaves	cooked
*Rumex obtusifolius *L.		HU	2	leaves	for compotes
*Rumex thyrsiflorus *Fing.		H	2	leaves	raw, cooked
*Rumex crispus *L.		L	1	leaves, seeds	flour, ff
*Polypodium vulgare *L.	*Polypodiaceae*	H	1,2,3	rhizomes	raw or cooked as sweetener
*Ranunculus ficaria *L.	*Ranunculaceae*	A	1, 3, 6	leaves	boiled, raw
*Nigella *sp.		L	1	seeds	spice
*Nigella arvensis *L.		A	6	seeds	spice
*Frangula alnus *Miller	*Rhamnaceae*	NU	2	fruits	jams
*Alchemilla *sp.	*Rosaceae*	L	1	leaves	boiled, ff
*Crataegus *sp. pl.		H	1, 2, 3, 5	fruit	raw, wine, jams
		A	6	flowers	raw
*Fragaria vesca *L.		H	1, 2, 3, 5	fruit	raw, wine, jams
*Malus domestica *Borkh. (feral plants)		O	1, 2, 3, 5	fruit	raw, compotes or in sauerkraut
*Potentilla anserina *L.		L	1	young shoots	raw
*Prunus avium *L.		O	1	fruits	raw, compotes
		O	1	solidified sap	raw
*Prunus padus *L.		H	1, 2, 3, 5	fruits	mainly raw
*Prunus spinosa *L.		H	1, 2, 3	fruits	raw, compotes, jams, wine
*Pyrus pyraster *(L.) Burgsd. *& Pyrus communis *L. em. Gaertner		O	1, 2, 3, 5	fruits	raw, dried, pickled
*Rosa *sp. pl. (mainly *Rosa canina L.)*		H	1, 2, 5	fruits	raw, wine, jams, infusions
*Rubus caesius *L.		H	1, 2, 3, 5	fruits	raw, wine, jams, infusions
*Rubus idaeus *L.		H	1, 2, 3, 5	fruits	raw, wine, jams, infusions
*Rubus *L. sect. Rubus sp. pl.		H	1, 2, 3, 5	fruits	raw, wine, jams, infusions
*Rubus saxatilis *L.		A	1, 2, 5	fruits	raw, juice
*Sedum maximum *(L.) Hoffm.		H	2	thick roots	unspecified
*Sorbus aucuparia *L. emend. Hedl.		H	1, 2, 3, 5, 6	fruits	wine, jams, liqueur, rarely as spice
*Galium odoratum *(L.) Scop.	*Rubiaceae*	H	2	flowering shoots	spice, infusions
*V. beccabunga *L. or *V. anagallis-aquatica *L.	*Scrophulariaceae*	A	3	shoots	raw
*Tilia cordata *Miller *& Tilia platyphyllos *Scop.	*Tiliaceae*	H	1, 2	flowers	infusion
		O	2	cambium	raw
		O	3	seeds	oil
		O	3, 4	leaves	boiled or into flour, ff
*Trapa natans *L.	*Trapaceae*	L	1	leaves	raw, boiled, flour
*Typha latifolia *L.	*Typhaceae*	A	5, 6	shoots and rhizomes	boiled, roasted
*Ulmus *sp.	*Ulmaceae*	N	2	fruits, leaves	unspecified
*Urtica dioica *L. *& Urtica urens *L.	*Urticaceae*	H	1, 2, 3	shoot tops	boiled, infusions
*Parthenocissus *sp.	*Vitaceae*	H	2	fruits	fermented drink
*Viola arvensis *Murr.	*Violaceae*	A	5	flowers	raw

The credibility of identification: H - confirmed by voucher specimen; A - confirmed by a reliable professional or amateur botanist; O - obvious common name universally used in a large area; L - probable Latin name or scientific name used in the language of a given country, given by a non-botanist; N - determined using comparative analysis of folk names; R - determined with the help of the data of a species range or/and habitat; U - highly uncertain; ff - used only as famine food. Source: 1 - [65], 2 - [79], 3 - [80], 4 - [81], 5 - [82], 6 - [83]

## Discussion

The lack of voucher specimens is obviously a problem in determining the real level of mistakes in older European ethnobotanical studies. A considerable number of errors was detected in the studied herbariums. The comparative analysis of species ranges and names did not reveal these mistakes. One may wonder to what extent other works can be trusted? Some ethnographers probably avoided making taxonomic mistakes by writing only about more common and widely known taxa and identifying taxa only to the genus level. Some authors mentioned in their publications that their study was documented by voucher specimens identified by a professional biologist (e.g. Orzeszkowa [77] and Wysłouchowa [64]) or that living/dried specimens were at least shown to professional botanists [30,46] or that voucher specimens from the Polish Ethnographic Atlas were used [46]. Thus in the above mentioned cases the possibility of mistakes is much lower.

Some taxa are more likely to be confused than others. Unsurprisingly, errors often occur in genera with more than one species, in which the species are similar to each other and are poorly recognized in folk taxonomy, e.g. in the genera *Mentha, Thymus, Tilia, Crataegus, Rosa, Rumex *and in the subgenus *Rubus*. The identification may be particularly difficult in apomictic taxa (like *Rubus *- [84]). Intergeneric mistakes occur either between two closely related genera not distinguished by folk taxonomy (*Carduus *and *Cirsium*) or less related (or unrelated) genera, if one of them has a folk name identical or similar to the scientific or folk name of another genus (in Poland, *Origanum *and *Chenopodium, Sonchus *and *Taraxacum*).

Nearly all of the quoted studies were performed by ethnographers, not botanists, so it is impossible to quantitatively compare the quality of their work with that of the few people with a biological background who have contributed to ethnobotany in Poland (e.g. Pirożnikow, Rostafiński, Maurizio, Moszyński, Szulczewski, though the latter two were known mainly as ethnographers). This comparison is particularly difficult given that most of these expert botanists supplied us with relatively large synthetic studies [66-69], and only Szulczewski and Pirożnikow contributed local monographs with ethnobotanical data [57,82]. Each of these studies [57,66-69,82] contains well over a hundred species. In five out of six of these works I have not encountered any identification mistakes. On the other hand in the work of Maurizio two mistakes can be suspected, which probably arose from the misidentification of folk taxa. Both concern famine plants used in Poland, quoted by the author. One of them is *Cichorium*, supposedly used as famine food in the Tatra mountains. Maurizio got this information from an ethnographic paper [25]. However the original source does not mention *Cichorium *but only a folk name - *szczerbak*. This folk name was used both for *Cichorium intybus, Cirsium rivulare*, as well as other *Cirsium *species [85]. C*irsium rivulare *was a very important famine plant in the Tatras [85], whereas *Cichorium intybus *was never mentioned as famine food by any other source listed in this article. Another possible mistake concerns the use of *Mellitis melisophyllum*. Maurizio mentioned that this plant (he also cited the folk name *miodnik*) was used during famine in Poland. However this is a relatively rare plant. On the other hand there are a few species of plants, which were used as famine food under similar names (*miodunka, medunka, miodownik*), i.e. *Lamium *spp., *Symphytum officinale *and *Pulonaria obscura *[65,79-81].

Even if these two mistakes were confirmed, the total ratio of mistakes in the works of the five professional botanists would remain well below half a percent. However, due to the different character of these studies, I restrained from deeper statistical comparisons.

It is worth pointing out that the quality of the ethnographers' work is highly variable. More than half of the publications contained no detectable mistakes, in contrast to a few authors who frequently misnamed the plants they had studied.

It must be noted that there is a significant difference between the mean percentage of mistakes detected in the studied works without voucher specimens (6.2%) and the level of errors found in the works documented by voucher specimens (9.2%). This also illustrates that even in works documented by a herbarium, gross mistakes can occur when the specimens are not verified by a good taxonomist. Single voucher specimens impose yet another threat: although the plant was correctly identified in the field or due to a widely known folk name a specimen of the wrong plant may be collected. This may have happened to Orzeszkowa. Her herbarium contains a specimen of *Hepatica nobilis *identified as 'kopytnik' *Asarum europaeum*. The name *kopytnik *is universally used throughout Poland to name *Asarum*, so Orzeszkowa *may *have collected a wrong specimen as both species have evergreen leaves of similar size and grow in the same habitat (identification scenario 1.1 or 1.2.2.1.2 in the Background chapter).

It must be emphasized that the main source of errors in the analyzed literature was the confusion of local names with Polish official names (scenario 2.1.3 in the Background chapter). This probably occurred by the researcher looking up a particular local name in a plant guide and then automatically assigning to it the Latin binominal of a different genus whose official name was identical to the local name of the studied species.

The studied papers usually contained little or no methodological information, so in most cases we cannot be sure if mistakes arose with or without seeing the actual studied plants in the field.

Most errors in the identification of voucher specimens occurred within the same genus, and only 24% of genera were misidentified. In contrast with this, 48% of mistakes detected in the publications without voucher specimens concern incorrectly identified genera. As the total number of detectable mistakes in the studies without voucher specimens is roughly four times lower than that of the studies with voucher specimens (2.3% versus 10%), we can assume that three quarters of all the errors and half of the misidentified genera remained undetected in the studies without voucher specimens.

It is a matter of dispute whether there should be separate codes for voucher specimens identified by an expert in the field and for those identified by a non-specialist (e.g. a separate code E could be used for a taxonomic expert). This could be useful, but on the other hand it is very difficult to draw a line between these two categories. As a rule, all voucher specimens should be identified/verified by a specialist - in case of easily identified taxa - a botanist, and in the case of critical taxa (in the Polish flora: *Rubus, Rosa, Hieracium, Oenothera, Alchemilla*) - a specialized taxonomist, or the specimens should be identified only to the genus, subgenus or section level [2-5,19].

Fortunately the errors made in the presented publications were rarely repeated later. The only case of erroneous "ghost information" in Polish studies is a list of plant names compiled in two ethnographic works [70,71]. This situation contrasts with Swedish publications, which according to Svanberg [8,9] contain numerous ghost data.

In all the studied cases the ethnobotanical herbaria contained species which had been reported in the given region or cultivated there, which supports the idea of using detailed atlases of plant distribution to verify ethnobotanical data. Such biogeographical data could be coupled with estimates of species abundance and distribution in local habitat spectra.

Not all the codes presented in the methodology section were used in the real-life list of edible plants of Poland. Most taxa were identified using the codes H, A and L, more rarely O, N or R. The codes D, M and P were not used. However this list was compiled using numerous voucher specimens (hence code H predominates) and data from 'reliable' researchers (like Rostafiński and Pirożnikow, hence code A). If a similar list were to be compiled for countries where voucher specimens were not collected, or for earlier periods, the proportion of codes in the list may have been reversed.

An interesting issue is the use of photography to document ethnobotanical studies [86]. Although photographic images cannot replace voucher specimens, they can help to document the use of plants, as many (but not all) taxa can be easily identified to a genus level from photographs [87]. Currently, as many electronic journals allow for the attachment of additional files to an article, authors could be encouraged to provide photographs of voucher specimens. Or perhaps we could start thinking about a service of online depositories of photographs of voucher specimens? Yet another option for plant identification, almost exclusively concerning historical ethnobotany, is the identification of plants from drawings (e.g. in old herbals). This is not always easy, but is sometimes possible, particularly when coupled with plant descriptions and folk names [73,88,89].

At the end of this paper the author must confess his own error. While preparing a table for the article on the taxonomic issues in Polish ethnobotanical studies [19], a mistaken name for *Veronica chamaedrys *was published, i.e. *wole oczy *instead of *żabie oczka*. ('ox-eyes' instead of 'frogs' eyes'). This mistake happened when transferring hand-written records to the computer. That is another example of the way errors and "ghost information" can enter ethnobotany, even via a botanist.

## Conclusions

Ethnographic papers without herbarium documentation contain on average at least 6.2% of mistakes. The verification of voucher specimens can increase this ratio to 9.2%. These mistakes most often arise by looking up plants using a local name in a botanical guide, and by the lack of cooperation between ethnographers and botanists. The large difference between the ratio of mistakes in the voucher specimen collections and the ratio of detectable mistakes in the literature is an argument for the rigorous use of voucher specimens, which are identified by a specialist, and for creating a service of online depositories of photographs of voucher specimens.

The presented code of credibility may become a useful tool for historical ethnobotany.

## Competing interests

The author declares that they have no competing interests.
